# Case Study of the Tensile Fracture Investigation of Additive Manufactured Austenitic Stainless Steels Treated at Cryogenic Conditions

**DOI:** 10.3390/ma13153328

**Published:** 2020-07-27

**Authors:** Róbert Bidulský, Jana Bidulská, Federico Simone Gobber, Tibor Kvačkaj, Patrik Petroušek, Marco Actis-Grande, Klaus-Peter Weiss, Diego Manfredi

**Affiliations:** 1 Department of Applied Science and Technology (DISAT), Polythecnic of Turin, V.le T. Michel 5, 15121 Alessandria, Italy; robert.bidulsky@polito.it (R.B.); federico.gobber@polito.it (F.S.G.); marco.actis@polito.it (M.A.-G.); 2 Kosice Self-govering Region, Namestie Maratonu mieru 1, 04266 Kosice, Slovakia; 3 Department of Plastic Deformation and Simulation Processes, Institute of Materials and Quality Engineering, Faculty of Materials, Metallurgy and Recycling, Technical University of Kosice, Vysokoskolska 4, 04200 Kosice, Slovakia; jana.bidulska@tuke.sk (J.B.); tibor.kvackaj@tuke.sk (T.K.); patrik.petrousek@tuke.sk (P.P.); 4 Institute for Technical Physics (ITEM), Karlsruhe Institute of Technology, 76131 Karlsruhe, Germany; klaus.weiss@kit.edu; 5 Department of Applied Science and Technology (DISAT), Polythecnic of Turin, Corso Duca degli Abruzzi 24, 10129 Torino, Italy

**Keywords:** additive manufacturing (AM), laser powder bed fusion (LPBF), 316L stainless steel, cryogenic treatment

## Abstract

Additive manufacturing is a key enabling technology in the manufacture of highly complex shapes, having very few geometric limitations compared to traditional manufacturing processes. The present paper aims at investigating mechanical properties at cryogenic temperatures for a 316L austenitic stainless steel, due to the wide possible cryogenic applications such as liquid gas confinement or superconductors. The starting powders have been processed by laser powder bed fusion (LPBF) and tested in the as-built conditions and after stress relieving treatments. Mechanical properties at 298, 77 and 4.2 K from tensile testing are presented together with fracture surfaces investigated by field emission scanning electron microscopy. The results show that high tensile strength at cryogenic temperature is characteristic for all samples, with ultimate tensile strength as high as 1246 MPa at 4.2 K and 55% maximum total elongation at 77 K. This study can constitute a solid basis for investigating 316L components by LPBF for specific applications in cryogenic conditions.

## 1. Introduction

At present, the manufacturing of complex-shaped parts, especially if made from difficult to work metallic alloys, is a true challenge in the field of manufacturing. Additive manufacturing (AM) technologies are currently considered to be a very promising solution to overcome such challenges, representing a quasi-pristine field of research for both metallurgists and process engineers. AM processes are a stimulating innovation, mainly in component design, enabling the manufacture of components not feasible via traditional methods [1,2].

AM shows the potential to substitute conventional metal manufacturing processes, such as casting and forging [3,4], mainly in the aerospace and biomedical fields. Thanks to improved reliability and reproducibility of the processes, AM competes with other progressive processes such as severe plastic deformation (SPD) [3,4,5,6,7] with substantial advantages such as the absence of constraints in the manufacturing design, shape freedom, high complexity of the components, combination of multiple parts into one part, production of functionally graded materials, reduced tooling requirements, and the possibility of production on demand [8,9,10].

The laser powder bed fusion process (LPBF), also known as selective laser melting (SLM), directly produces homogenous metal objects, layer by layer, from 3D Computer Assisted Drawing (CAD) data, by selectively melting fine layers of metal powder with a laser beam [1,2,3,4,5,6,7,8]. A considerable amount of material can be saved by designing properly for additive manufacturing with no loss in terms of mechanical properties [11,12,13].

Austenitic stainless steels (especially the grade 316L) have been studied deeply in the conventional cast/wrought grades, but also in the field of AM it has been among the first materials to be widely processed and thus the object of many literature studies [14,15]. Its properties and the very low amount of carbon make this material suitable for welding, whose metallurgical mechanisms (laser welding) are the basis for understanding laser-based additive manufacturing techniques such as laser powder bed fusion (LPBF). In terms of application, 316L stainless steel finds broad application as a structural material for cryogenic temperatures below 77 K, considering its relatively high strength at low temperatures (>1200 MPa). This is the result of the face-centered cubic (fcc) atomic structure of the austenite, as derived by alloying with nickel [16]. Subjecting steels to cryogenic treatment to improve their properties was conceived since modern technologies employ many devices operating at very low temperatures. Cryogenic treatments are a relatively new process mainly devoted to carbon alloyed steels to eliminate retained austenite. In order to perform cryogenic treatments, the temperature has to be lowered for metallurgical transformations to occur (e.g., transformation of retained austenite to martensite). Significant improvements have been documented for AM 316L as well, under wear testing conditions [17].

Villa and Somers [18] underline that despite many investigations, the metallurgical understanding of the microstructural changes involved in the cryogenic treatment of steel has remained poor. Especially, in AM products, cryogenic treatment is still very rare. The austenitic 316L grade is a popular steel for cryogenics as demonstrated by the vast literature, with special applications in the field of liquid gas confinement and storage [1,2,3,4,5,6,7,8,9,10,11,12,13,14,15,16,17,18,19,20,21,22,23,24]. Mechanisms governing tensile properties at cryogenic temperatures for wrought materials have been extensively and deeply investigated [25]. In metastable stainless steels like 316L, the austenite transforms to martensite when plastic deformation occurs. Martensite is far more stable in those conditions thus increasing the material’s work-hardenability. Parameters such as stacking fault energy, temperature, composition of the alloy, and plastic strain affect the lattice of the formed martensite either as hexagonal close-packed or body-centered cubic. The dynamic recovery of dislocations is hindered at low temperatures for austenitic stainless steels, such aspect leads to an increase in the density of twins, dislocations, and plastically-induced martensite. At this point the mechanical behavior is characterized by a second hardening after yielding. This mechanism is attributable to the transformation-induced plasticity mechanism (TRIP). AM technologies are very promising thanks to their distinctive feature of allowing complex near net shapes to be manufactured almost effortlessly. At the moment, the literature completely lacks in the characterization of tensile properties of 316L in cryogenic conditions. Despite such enormous potentiality, AM techniques are still considered as novel manufacturing processes and as a result, literature is continuously updating on this subject. To the authors’ best knowledge, no study has been carried out to characterize tensile properties of LPBF 316L under cryogenic conditions.

In order to understand the metallurgical considerations governing cryogenic steels, it is useful to focus on the basic mechanical properties’ yield strength (YS), ultimate tensile strength (UTS), and toughness, or uniform elongation (UE). Both strength and toughness are critical properties, since failure may occur through either ductile rupture or fragile fracture. Their combination is important since strength and toughness have an inverse relation to one another; an increase in strength at a given temperature almost invariably leads to a decrease in toughness. Hence, the present paper focuses on the fracture mode and the tensile properties of 316L stainless steel processed through LPBF, tested at cryogenic temperatures. LPBF generally creates a high residual stress state in the produced components, resulting in distortion and warping, leading to a variation of the final mechanical properties. Stress relieving, usually performed right after the AM processing, helps in reducing residual stresses, increasing ductility and decreasing tensile strength.

## 2. Materials and Methods

The gas atomized 316L stainless steel powder (produced by Electro Optical Systems (EOS, Krailling, Germany) was used as experimental material. The chemical composition provided by the supplier is reported in Table 1. The extremely low concentration of S and P, together with the concentration of alloying elements in the upper bound of their acceptance intervals (Cr, Ni, Mo), confer to this composition an austenitic microstructure at room temperature and a high chemical resistance. Sieve analysis was performed according to practices described in the Association for Standard and Testing of Materials standard ASTM B 214-07 (using a Retsch AS200 Digit sieving system (Retsch, Haan, Germany) following the ASTM E11 standard.

The specimens for the static tensile test were prepared by LPBF technology by EOSINT M270 Dual Mode machine (EOS, Krailling, Germany), equipped with a 200 W Yb fiber laser. The process parameters indicated in Table 2 were adopted.

**Figure 1:**
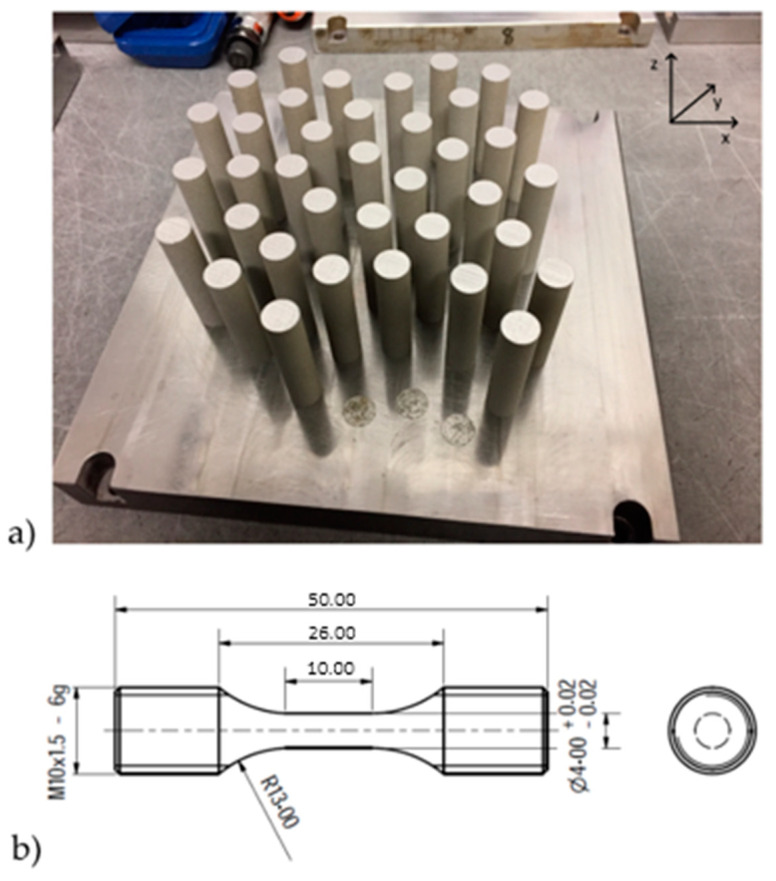
(**a**) Base plate with as-built samples just after the LPBF printing process and (**b**) mechanical drawing of the samples manufactured for tensile testing.

**Figure 2:**
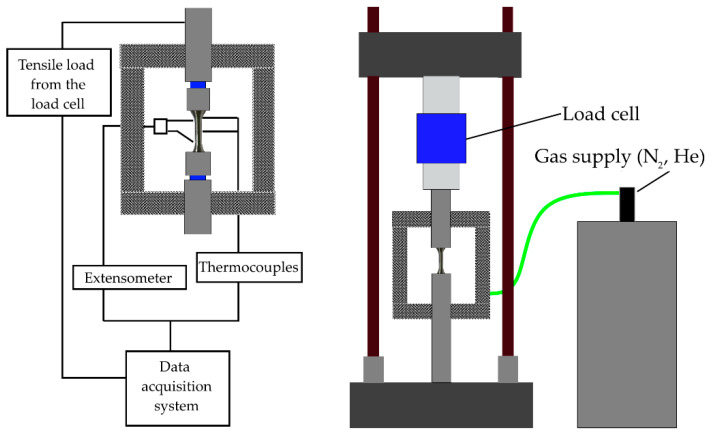
Test setup for tensile testing of steel samples under cryogenic conditions.

After manufacturing (Figure 1), the samples were divided into two sets. The first set of samples was labeled “as-built” while another set was heat-treated by means of stress-relieving to promote the relaxation of retained stresses building up during the AM process. The heat treatment parameters for stress-relieving were 400 °C and 60 min soaking time. Such stress-relieving treatment was carried out in a vacuum furnace (TAV MiniJet HP 235) (TAV, Caravaggio, Italy) with a vacuum level approaching 9 × 10^−3^ mbar. Due to the low temperature employed, there was no need for Ar backfilling to prevent degassing of elements with low vapor tension. Microstructural analysis was performed on samples in the as-built condition; samples were cut and mounted in phenolic resin (Presi, Eybens, France) to observe the microstructure along the building direction (*z*-axis) and on the scanning plane (xy-plane). After mounting in resin, the samples were grinded with abrasive SiC-based papers (400, 800, 1200, 2400 grit) (Presi, Eybens, France) to and then polished with cloths soaked with diamond suspension (3 µm and 1 µm average size) (Presi, Eybens, France). Metallographic samples were observed in the as-polished condition to evaluate the average residual porosity by image analysis software Image J (National Institute of Health, Maryland, MD, Bethesda, USA) and then etched with aqua regia to observe the microstructure by scanning electron microscopy. The static tensile test was carried out on samples at three different temperature conditions: 298 K, 77 K, and 4.2 K. The device used was the MTS100 Landmark (MTS, Eden Prairie, Minnesota, MN, USA) equipped with a cryostat and extensometer. At least three specimens for each condition were tested. Tests were performed according to the ASTM E8M standard. The cryostat with a vacuum shield was used for the cooling down process to liquid nitrogen and helium temperature (Figure 2). Fracture analysis was carried out by field-emission scanning electron microscope (FE-SEM; Zeiss EVO) (Zeiss, Oberkochen, Germany) equipped with energy dispersive X-ray analysis (EDS) (Oxford Instrument, Abingdon-on-Thames, UK). The average size of dimples was evaluated by means of the linear intercept method (according to ASTM E112-96) [26,27]. Different areas were identified as the fracture surface, the sum of which is considered to be representative of the investigated material, by measuring microstructural features on different micrographs. However, due to the non-flat topography of the fracture surface, the chosen approach caused a reduction of the investigated zones to those perpendicular to the detector of the FE-SEM, considering that the analysis of non-perpendicular planes would cause a non-correct evaluation of the dimples. Therefore, only dimples showing a spherical shape were considered, taking into account that the shape would be the result of an analysis perpendicular to the void. The determination of the average diameter was carried out through the evaluation of no less than 500 dimples.

Further analyses were performed on fracture surfaces to evaluate the percentage of ductile fracture. A ductile fracture is characterized for showing a diffuse coalescence of dimples; such dimples are responsible for the plastic deformation occurring in the material. Nevertheless, in conventional powder metallurgical (PM) products, fracture paths and fracture resistance are related to details of the complex, locally inhomogeneous, microstructures with pores and weak interfaces. All these have characteristic fracture resistance properties resulting in combinations of dimple rupture, cleavage, and intergranular and interparticle failure micromechanisms. Based on the criteria of previous studies (see [28,29]), the fracture surfaces of AM 316L samples were evaluated. Ten SEM images at 3000× magnification of the fracture surface of each sample were analyzed via the image analysis software (Image J) to calculate the fraction of dimples showing coalescence. The dimples grown together are those responsible for load-bearing capacity of the cross-section. These data together with dimple size calculation can explain from fractography the results observed by mechanical testing.

## 3. Results and Discussion

**Figure 3:**
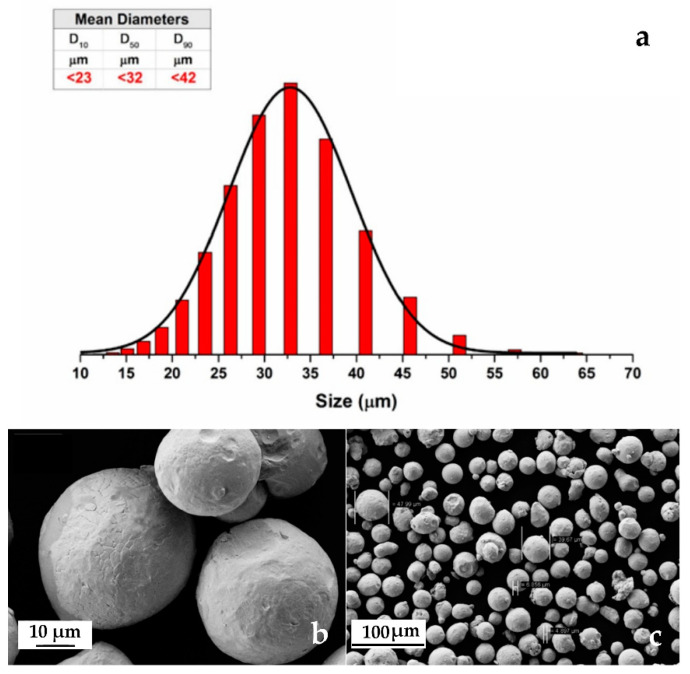
(**a**) Granulometry results with table and graphical interpretation; (**b**) detail of 316L stainless steel powders (FE-SEM) and (**c**) the same powders at low magnification (FE-SEM).

Conventional powder metallurgy (PM) research often underlines the benefits of tailoring specific powder properties, through simple steps, to allow significant improvements in the properties of the sintered component and to increase efficiency during powder processing [30,31,32]. The results of particle size and shape are presented in Figure 3.

**Figure 4:**
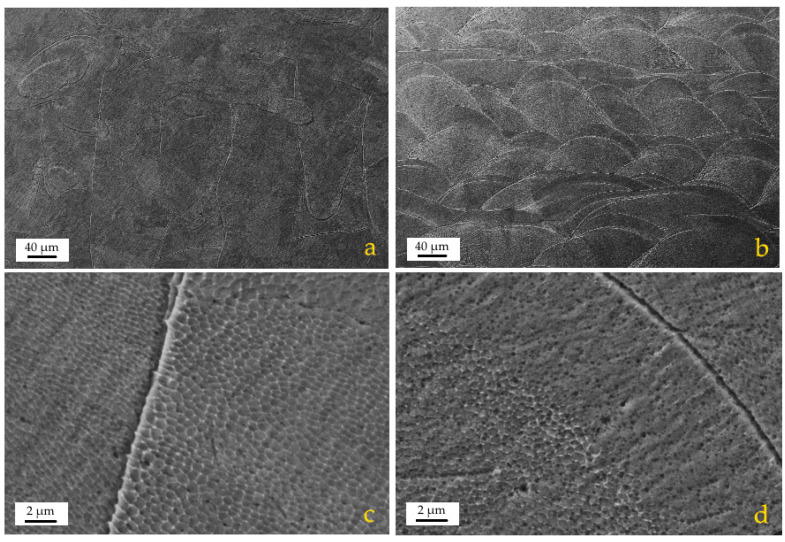
FE-SEM images of the as-built microstructure after etching: (**a**) overview parallel to the xy plane; (**b**) overview along the z axis (building direction); (**c**,**d**) magnified micrographs of (a) and (b), respectively.

From Figure 3 it is clear that the investigated powders have a spherical shape (Figure 1b) with a mean diameter of 32 μm (Figure 1a). Figure 3c shows that atomized 316L stainless steel powders consist of different sizes, starting from around 5 μm up to 50 μm. The granulometric results show that the mean diameter of the investigated powders was 32 μm, as presented in Figure 1, if the distribution is made by volumetric assumption. The AM samples have high density (>99.5%) and the typical microstructure of LPBFed materials. A substantial microstructural anisotropy between the laser scanning plane xy (Figure 4a) and the building direction z (Figure 4b) is characteristic for AM 316L stainless steel [15]. At higher magnification the complex sub-micron cellular microstructure and fine sub-grains in the large grains are evident (Figure 4c,d). Similar considerations to those found in literature [15] can be applied to the description of the microstructures from the samples of this study. Very little defectiveness of the samples was observed in terms of lack of fusion and porosity; such an observation is significant for the successful choice of optimal process parameters.

A summary of the mechanical properties is presented in Table 3. The total elongation (TE) at fracture and the total area of reduction at fracture (RA) are considered to be provide indications of material ductility. However, the UE better represents the material’s ductility or formability in uniaxial deformation, because after necking (when uniform elongation is exhausted), the material can be considered to have failed. The testing temperature has a significant effect on mechanical properties; in fact, it can be addressed as the main factor affecting the variation in mechanical properties between the as-built and the stress-relieved series of samples. On the contrary, no relevant variation in mechanical properties is attributable to stress-relieving heat treatment. Both YS and UTS increase with the reduction of the testing temperature; the reasons for such behavior have already been investigated deeply in the literature [25] for casting/wrought 316L and are related to the TRIP properties of this alloy. The findings reported in Table 2 confirm, for the first time, that AM 316L in cryogenic conditions also follows the same behavior as the alloy obtained from secondary metallurgy processes. The large cooling rate is responsible for producing a part with a finer grain microstructure and better tensile properties than those made of the traditional wrought counterparts, as can be compared to the study [33] for 316L stainless steel cast microstructures). The results obtained in the frame of this paper, in terms of relevant ductility, are provided by values of good elongation and high yield strength. According to different authors [34,35], high YS is due to the high dislocation density in LPBF steel. In terms of tensile properties, both YS and UTS strongly increase with temperature decreasing from 298 to 4.2 K: YS increased from 500 to around 800 MPa and UTS increased from around 560 to over 1200 MPa. The degree of ductility varies with testing temperature too, but concerning load-related properties such as YS and UTS, its behavior is not monotonic.

**Figure 5:**
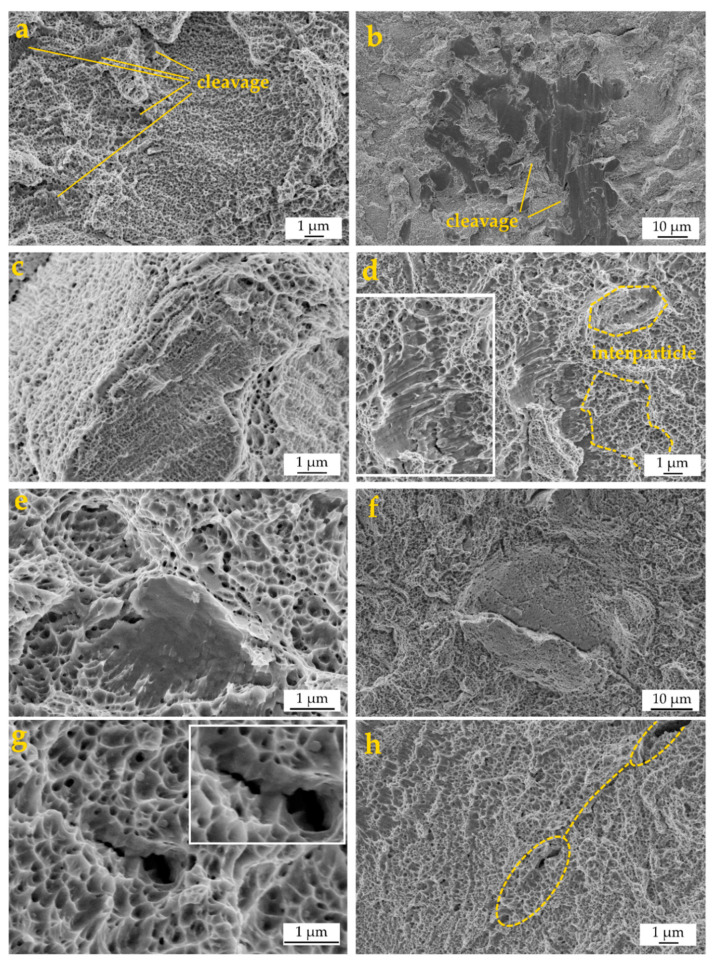
Example of principal features on the fracture surface of an LPBF 316L tensile specimen: (**a**) ductile fracture; (**b**) cleavage; (**c**) interparticle fracture; (**d**) cleavage cliff in the ductile region; (**e**) cleavage facet surrounded by ductile dimples, (**f**) microcrack growth obstructed by high toughness regions; (**g**) microcrack–microstructure interactions; (**h**) pore-nucleated microcrack.

Both the elongation, either UE and TE, and the RA are representative of materials’ ductility, but from the results presented in Table 2 it is evident that they do not follow the same trend with decreasing temperatures. The RA decreases with decreasing temperatures while both UE and TE show a maximum at 77 K. The TE at 298 K is coherent with other results found in the literature [14,15] for AM 316L stainless steel, while for lower temperatures it is not possible to make a direct comparison with additively manufactured products due to the complete lack of studies in this field. A comparable dissimilarity between the trend of elongation and that of the RA was observed by Crivoi et al. [36]. They attributed the difference in the trend between elongation and RA to the experimental error in measuring the elongation with the tensile testing machine’s cross-bar. The elongation values presented in Table 3 were obtained after measuring with an extensometer and, furthermore, were reproduced in several samples at two different conditions: as-built and stress-relieved. After this consideration it is reasonable to suppose the effect of either systematic or casual error to be mitigated. Despite being the focus of this article on fractography, some hypothesis can be raised to explain the different trend between elongation and the reduction of area. UE is the value representative for the elongation before the occurrence of localized necking in the sample. An increase in length with temperature decreasing from 298 to 77 K is supposedly due to the formation of martensite in the austenitic microstructure, enhancing YS and resistance to necking. After necking of the sample at cryogenic temperatures (77 K and 4.2 K), further plastic deformation is minimal and consequently so is the difference between TE and UE, below 1%. The samples tested at room temperature have lower YS and for this reason necking occurs after just 4% elongation, but elongation progresses until reaching a TE of 35% at rupture, with 31% difference between TE and UE. The whole deformation occurring after necking is localized predominantly in the neck area and for this reason the highest RA is measured at 298 K; at this temperature the sample during tensile testing undergoes high deformation after necking. At cryogenic temperatures the measured RA almost corresponds to that which is measurable at the onset of necking. At 77 K the sample exhibits a level of UE higher than 50% distributed along the whole gauge length, while at 4.2 K such deformation is between 28% and 36%. The measurement of RA is performed in correspondence to the thinnest part of the gauge length. The large difference between UE and TE, 5% vs. 35% respectively, means that after the occurrence of necking, the material still deforms plastically before breaking; such observation is supported by the value of RA, almost 50%. The samples tested at room temperature undergo a progressive cross-sectional shrinkage, and then break after reaching a TE of approximately 35%. Samples tested at cryogenic temperatures experience a decrease in the RA value which is not coupled to a decrease in elongation that, on the contrary, increases significantly (56% at 77 K and 28% at 4.2 K). Considering the samples tested at room temperature, UE and TE values almost tend to overlap; the localized necking mechanism is reduced, favoring a more uniform necking along the length of the sample as revealed by the RA value. The hypothesis for such behavior can be related to the balance between martensite formation and dislocation movement inhibition at low temperatures. On one hand, martensite formation occurs intensively at cryogenic temperatures both in the first and in the second hardening range [36]; this mechanism would explain the increase in mechanical resistance at cryogenic temperatures. On the other hand, the movement of dislocation is hindered at cryogenic temperatures and consequently plastic deformation is reduced as soon as necking occurs. Fracture surfaces were observed by FE-SEM and their most representative features are reported in Figure 5.

The fracture surfaces exhibited a mixed morphology with both particle necks, characteristic of ductile fracture (Figure 5a) and cleavage, which is more evident at lower temperatures (Figure 5b). The fracture path is mixed, both inter and intra particle (Figure 5c), and in the same area very different behaviors can coexist (Figure 5d). Typical features of fragile fractures, such as cliffs and quasi-cleavage, are found inside the ductile region, showing a very distinctive aspect (Figure 5d,e).

FE-SEM images from Figure 5f–h are representative of some microstructure–fracture interactions: in Figure 5f a microcrack was detected, seemingly originated from a microstructural defect such as a microporosity, whose growth is obstructed by the surrounding high toughness region, diffused dimples; in Figure 5g, the microcrack–microstructure interactions with detail of microcrack growth are obstructed by high toughness regions; in Figure 5h, the pore-nucleated microcrack in the yellow ellipse grew and coalesced with a neighboring, pore-nucleated microcrack also in a yellow ellipse close to the border of the picture. A high volume of the fractures corresponds to the typical features of ductile materials, with small dimples less than 250 nm in diameter, visible throughout the surface (Figure 5a). The dimple size is smaller than that obtained in previous studies on steels processed by press and sintering [37,38]. A reason may be found in the refined cellular structure which affects the nucleation and growth of micro-voids. However, a few larger dimples around 1 μm in diameter were also detected.

**Figure 6:**
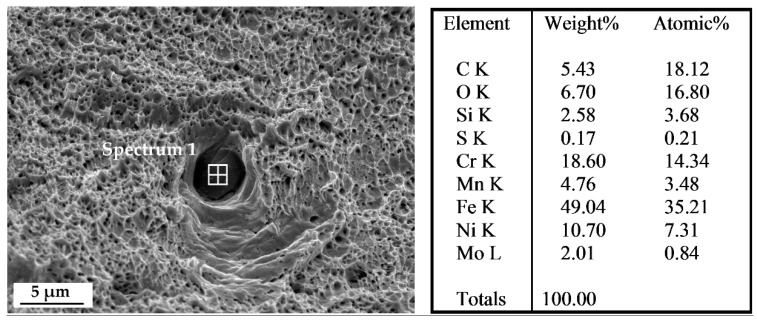
FE-SEM micrograph with an example of Cr-Mn-Fe inclusion, as demonstrated by EDS analysis.

On one hand, the inclusions observed in Figure 6 act as stress concentrators thus facilitating the occurrence of cleavage and brittle fracture micro-mechanisms in the surrounding area. On the other hand, very few partially molten particles (i.e., lack of fusion) were detected on the fracture surfaces; such evidence confirms the successful processing of the powders to a solid with density close to the theoretical one.

**Figure 7:**
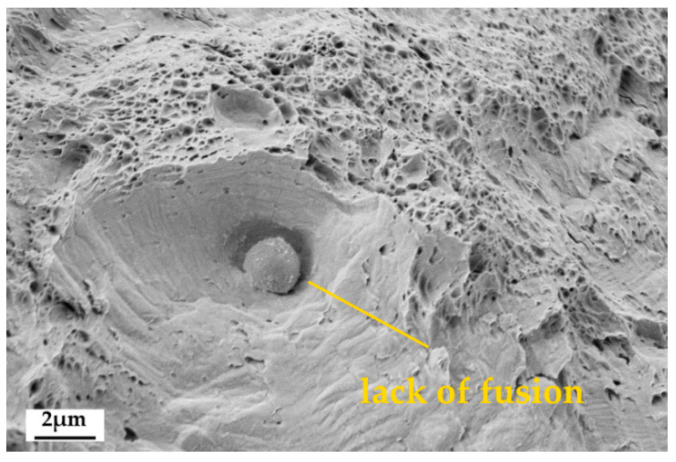
FE-SEM image of a partially molten steel particle on the fracture surface.

As observed in Figure 7, there are no dimples in the area surrounding the lack of fusion. The area around the lack of fusion fractures by typically brittle mechanisms, such as cleavage, due to the premature debonding of partially molten particles that might start at low-stress levels thus locally reducing the load bearing section of the sample. Microstructural defects can have a significant role in reducing mechanical properties in terms of strength and ductility; for this reason the presence of such defects must be kept under control. The proper tuning of the process parameters is responsible for minimizing the occurrence of fusion, but residual porosity, for instance, is a defect related to powder quality too, much like non-metallic inclusions. Obtaining a fully dense and defect-free material is a non-trivial issue in AM: the main LPBF process parameters together with starting powders characteristics, as chemical composition, mean size, and sphericity are relevant factors and interact at different levels.

## 4. Discussion

Metal AM is characterized by high temperature gradients, high cooling rates, and cycling reheating [9,10], causing large microstructure differences from traditional manufacturing routes [11]. This definition is largely adopted to justify the variation in microstructural and mechanical properties observed in AM alloys [39].

Nevertheless, microstructural defects can arise from the LPBF additive technique that is defined according to three categories: spherical porosities, irregularly incomplete fusion holes, and cracks. Spherical porosities are randomly distributed, while incomplete fusion holes are generally distributed between the tracks and layers. Such defects can be responsible for crack initiation or bridging as they are discontinuities inside the material or stress concentrators.

**Figure 8:**
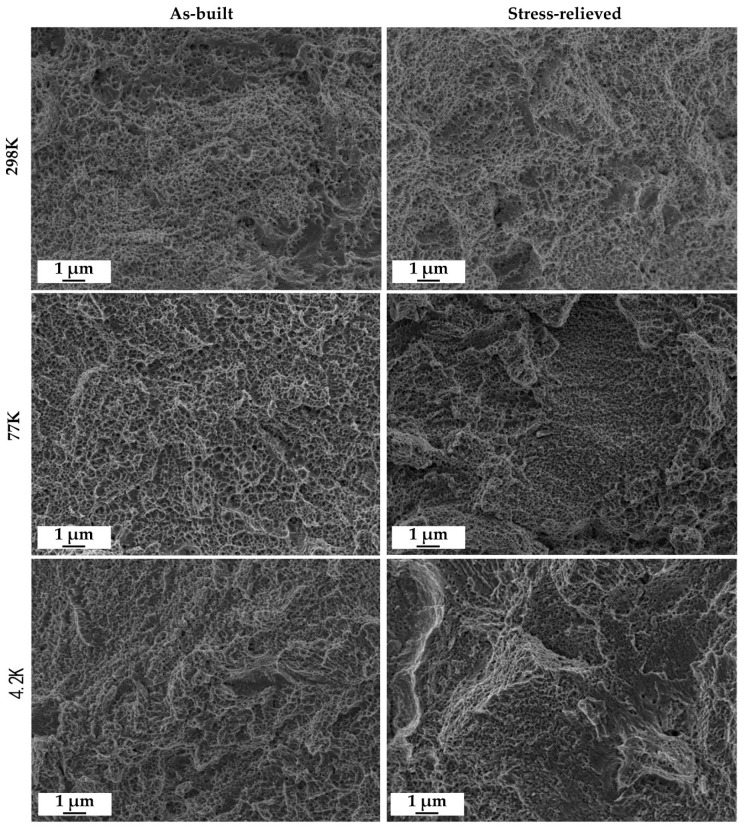
Representative FE-SEM images of the fractography used for quantitative image analysis.

**Figure 9:**
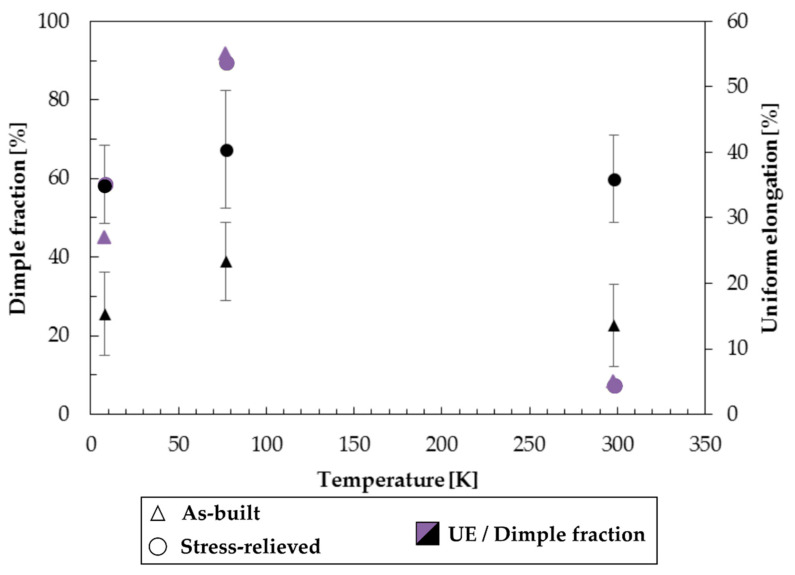
Results from quantitative image analysis: dimple average fraction across the fracture surface as a function of temperature for the 316L samples by LPBF tested in the as-built and stress-relieved conditions.

To discuss the correlation between mechanical and microstructural properties for the specific case of AM 316L tested at cryogenic temperatures, the average size of dimples (Table 4) and the number of dimples across the fracture surface were observed directly on the fracture surfaces and measured by image analysis (Figure 8 and Figure 9). In Figure 8 the general overview of fracture surfaces for the different testing conditions is reported. As for the literature, an increase in dimple size is the response given by the fracture surface to the increase in tensile strength and ductility [40]. The dimple size increasing with decreasing temperature, as reported in Table 4, is associated with the increase in both UTS and UE; despite the maximum UE observed for 77 K, this cannot be explained from the sole dimple size/dimple fraction measurement but would need further, deep microstructural investigations to assess the kinetics of martensite formation during tensile testing. From the data analyzed, it was found that the stress-relieving treatment does not have a significant effect on ductility for 316L stainless steel while tensile properties tend to be slightly higher for the material in the as-built condition.

The austenitic steels that are commonly specified for cryogenic service are purified of metalloid impurities and, in the case of the Fe-Mn grades, lightly alloyed with beneficial grain boundary surfactants (C and Si). They are then solution strengthened with interstitial nitrogen. If the alloy has significant concentrations of Cr and Mn, the nitrogen solubility is high, and nitrogen concentrations of 0.2–0.4 wt.% can be added without embrittling grain boundaries. As a consequence, these alloys are not subject to brittle fracture, even when hardened to a very high strength at 4.2 K [41].

From a general point of view, it is well established in the literature that austenitic steels, such as 316L, show transformation-induced plasticity characteristics (TRIP); such a behavior is supposed to be responsible for the increase in UTS observed with decreasing temperature (Table 1). In addition, a toughening effect is associated with twinning, while the extremely-fine grain size provided by LPBF is another factor affecting both toughness and strength. The explanation for increased UTS at cryogenic temperatures has to be searched within the interaction of such factors, competing with the reduction in strength due to the development of pores and the presence of defects induced by the manufacturing technology itself. Consequently, deformation twinning can lead to significant ductility but does not result in a high hardening rate of the AM 316L.

Due to the still low number of studies on microstructures after deformation, it is still unclear why AM 316L has a good elongation, which is reported to be as good as that made by other processes [9], despite some residual porosity and the high residual stresses in AM samples [6].

Comparable results have been found (see [42]) when testing laser welded joints of 316LN at cryogenic conditions at 4.2 K. The principle of LPBF can be considered the same when applied in laser welding. The comparison of fracture surfaces between this work and previous research [35] shows numerous similarities in terms of morphology and mechanical properties. The fracture surfaces are characterized by ductile fracture features with multiple dimples and microvoids, which result in excellent mechanical properties of both the welded joints of a previous study [42] and the AM samples of this study. The dimple fraction reported in Figure 9 is an indicator of ductility at the microscale, and despite a relevant uncertainty in the measured data, its trend is in accordance with the UE one.

## 5. Conclusions

LPBF was employed to produce 316L stainless steel samples for tensile tests. The samples were tested in two conditions, as-built and after a stress-relieving heat treatment. Three temperatures were chosen for tensile tests: ambient temperature, and two cryogenic temperatures, 77 K and 4.2 K. The main findings are summarized below:

- The main factor impacting the mechanical properties of the tested samples was the testing temperature, while the stress-relieving treatment did not significantly affect the results both in terms of mechanical strength and ductility in comparison to the as-built condition. High tensile strength at cryogenic temperatures was characteristic for all samples, with UTS reaching its maximum value of 1246 MPa at 4.2 K. At ambient temperature, the UTS was about 560 MPa for both as-built and stress relieved conditions.
- Very high elongation at failure was characteristic of the samples tested at cryogenic temperatures. At 77 K the highest TEwas reached, being 53% for as-built and 56% for stress-relieved samples. While at 4.2 K, TE was 36% for as-built and 26% for the stress-relieved samples respectively.
- FE-SEM analyses indicate the formation of macro and microcracks over the entire surface after the static tensile test at 4.2 K, with a dimple fraction that was directly proportional to the level of UE reached.

## Figures and Tables

**Table 1:** Chemical composition of 316L powder as reported by supplier technical datasheet.

Elements	C	Cr	Ni	Cu	Mn	Si	Mo	S	P	N	Fe
wt.%	0.009	18.20	13.98	<0.005	1.68	0.32	2.79	<0.005	0.015	0.07	Bal.

**Table 2:** Process parameters adopted for the production of of 316L stainless steel samples by LPBF.

Power (W)	Layer Thickness (µm)	Scanning Speed (mm/s)	Hatching Distance (mm)	Temperature of Building Platform (°C)
195	20	800	0.09	80

**Table 3:** Mechanical properties after tensile testing of 316L samples by LPBF at three different temperatures (298 K, 77 K, 4.2 K) in the as-built or stress-relieved state.

Conditions	Testing Temperature(K)	YS ^1^ (MPa)	UTS ^1^ (MPa)	UE ^1^ (%)	TE ^1^ (%)	RA ^1^ (%)
As-built	298	499 ± 12.1	564 ± 15.4	4 ± 2.7	35 ± 3.4	49 ± 2.1
	77	726 ± 17.7	1083 ± 36.2	53 ± 7.4	53 ± 7.9	23 ± 3.4
	4.2	802 ± 23.5	1246 ± 42.6	35 ± 3.2	36 ± 4.1	16 ± 1.1
Stress-relieved	298	500 ± 31.2	565 ± 19.4	5 ± 3.1	18 ± 6.2	48 ± 4.5
	77	730 ± 17.6	1080 ± 29.3	55 ± 6.4	56 ± 8.5	24 ± 3.1
	4.2	805 ± 32.4	1200 ± 34.2	27 ± 4.7	28 ± 3.2	15 ± 2.3

^1^ YS—yield strength; UTS—ultimate tensile strength; UE—uniform elongation; TE—total elongation; RA—reduction of area.

**Table 4:** Average dimple size for the measured 316L samples in two conditions after tensile testing at different temperatures.

Temperature (K)	As-Built Average Dimple Size (µm)	Stress-Relieved Average Dimple Size (µm)
298	0.28 ± 0.05	0.24 ± 0.03
77	0.41 ± 0.02	0.28 ± 0.04
4.2	0.48 ± 0.07	0.34 ± 0.05
